# Quality Control Methods in Accelerometer Data Processing: Identifying Extreme Counts

**DOI:** 10.1371/journal.pone.0085134

**Published:** 2014-01-13

**Authors:** Carly Rich, Marco Geraci, Lucy Griffiths, Francesco Sera, Carol Dezateux, Mario Cortina-Borja

**Affiliations:** Medical Research Centre of Epidemiology for Child Health, University College London, London, United Kingdom; NIDDK/NIH, United States of America

## Abstract

**Background:**

Accelerometers are designed to measure plausible human activity, however extremely high count values (EHCV) have been recorded in large-scale studies. Using population data, we develop methodological principles for establishing an EHCV threshold, propose a threshold to define EHCV in the ActiGraph GT1M, determine occurrences of EHCV in a large-scale study, identify device-specific error values, and investigate the influence of varying EHCV thresholds on daily vigorous PA (VPA).

**Methods:**

We estimated quantiles to analyse the distribution of all accelerometer positive count values obtained from 9005 seven-year old children participating in the UK Millennium Cohort Study. A threshold to identify EHCV was derived by differentiating the quantile function. Data were screened for device-specific error count values and EHCV, and a sensitivity analysis conducted to compare daily VPA estimates using three approaches to accounting for EHCV.

**Results:**

Using our proposed threshold of ≥ 11,715 counts/minute to identify EHCV, we found that only 0.7% of all non-zero counts measured in MCS children were EHCV; in 99.7% of these children, EHCV comprised < 1% of total non-zero counts. Only 11 MCS children (0.12% of sample) returned accelerometers that contained negative counts; out of 237 such values, 211 counts were equal to −32,768 in one child. The medians of daily minutes spent in VPA obtained without excluding EHCV, and when using a higher threshold (≥19,442 counts/minute) were, respectively, 6.2% and 4.6% higher than when using our threshold (6.5 minutes; *p*<0.0001).

**Conclusions:**

Quality control processes should be undertaken during accelerometer fieldwork and prior to analysing data to identify monitors recording error values and EHCV. The proposed threshold will improve the validity of VPA estimates in children’s studies using the ActiGraph GT1M by ensuring only plausible data are analysed. These methods can be applied to define appropriate EHCV thresholds for different accelerometer models.

## Introduction

Accelerometers are widely used in large-scale studies to objectively measure children’s physical activity (PA). This is particularly important since subjective methods such as self- and parent-proxy reports often overestimate PA levels [1]. Accelerometers measure time-varying differences in movement of body mass, and can be used to assess the frequency, intensity, and duration of PA. Technological advances have made accelerometers smaller, lighter, and less expensive. These developments, combined with increased battery life and memory storage, have made accelerometer data collection feasible in population-based studies in children. However, uncertainties remain regarding the implementation of accelerometer data processing protocols.

ActiGraphs (ActiGraph, Florida, USA) are the most commonly used accelerometers in children’s PA research, as they are small (3.8×3.7×1.8 cm), lightweight (27 g), and have been extensively validated [2], [3]. The ActiGraph GT1M [4], [5], [6] is favoured over the older 7164 model [4], [7], [8] due to its increased battery life and memory storage, and also because it does not require calibration after it leaves the factory [9], [10], [11]. Both devices are designed to record accelerations (recorded as ‘counts’) within a defined range of movement for humans. Signal frequency filtering techniques are employed within these models to exclude accelerations unlikely to be generated by human movement, such as those caused by electrical noise and mechanical vibrations. The firmware digital filter in the GT1M is favoured over the more error-prone hardware digital filter in the ActiGraph 7164 [12]. Despite these filtering systems, extreme high count values (EHCV) have been recorded by the 7164 model, including repeated values equal to 2^15^ −1  =  32,767 which indicate voltage signal saturation within the monitor, at which value the accelerometer malfunctions.

Previous authors have emphasised the need for quality control procedures that may result from monitor malfunctions or participant tampering [9], [13]. In order to identify EHCV, a threshold is needed that is low enough to exclude EHCV, but high enough to include genuine records of vigorous PA (VPA). Only a few studies have used [9], [14], [15], [16], [17], and even less have calculated [15], [16], [17], [18] a threshold to remove EHCV. In those that have calculated a threshold, the value is based on data collected using the ActiGraph 7164 and/or using conservative statistical methods. It is not known what influence including EHCV has on estimates of VPA. This may threaten the validity of activity estimates, potentially introducing biases and increasing measurement errors. In the absence of a standardised threshold to remove EHCV it may be difficult to compare PA data across studies using the same accelerometer model. Even small differences in data processing can have a substantial impact on derived outcome variables [19], [20], [21]. In addition, no previous studies have reported whether the ActiGraph GT1M records the same typical error count values as the 7164.

We therefore carried out a study aiming to: firstly, develop methodological principles for establishing an EHCV threshold, and propose such threshold for the ActGraph GT1M using population-based accelerometer data in UK children; and secondly, determine (i) typical error values recorded by the ActiGraph GT1M and the consistency of pre-defined accelerometer initializing parameters (e.g. start time and date, epoch mode); (ii) frequency of EHCV; and (iii) the influence of varying the approach to determine EHCV on daily estimates of VPA.

## Methods

### Data collection

Population-based accelerometer data obtained as part of the Millennium Cohort Study (MCS) were used in all statistical analyses. The MCS is a longitudinal UK-wide study of children born in the new century sampled to ensure an adequate representation of all four UK countries, disadvantaged areas, and ethnic minority groups [22], [23]. At age seven years, accelerometers were used to measure children’s PA levels. All children participating in the MCS at this age were invited to wear an accelerometer and written consent obtained from parents/guardians of those agreeing.

Children were asked to wear the ActiGraph GT1M (15 second epoch) on an elasticated belt on their right hip for seven consecutive days during all waking hours, except during bathing or swimming. Accelerometers were posted to families who were asked to return it as soon as possible after the monitoring period using a supplied pre-paid envelope. Accelerometers were distributed between May 2008 and August 2009.

The MCS accelerometer study was approved by the Northern and Yorkshire Research Ethics Committee (REC number: 07/MRE03/32). The MCS accelerometer data (SN:7238) are currently available via the UK Data Service.

### Statistical analysis

Accelerometer data were downloaded using ActiLife Lifestyle Monitoring software (version 3.2.11), and were processed using algorithms developed in the R software environment for statistical computing (version 2.15.0) [24]. Data from all MCS children who returned an accelerometer were included in our analyses unless parents/guardians had explicitly stated that the accelerometer had not been worn (*n* = 9005).

To define an EHCV threshold we developed a procedure based on the sample quantile function of the accelerometer counts restricted to positive values only. Firstly, we estimated the empirical quantile function for a fine sequence of probabilities 𝛕, namely from 𝛕  = 0.8 to 𝛕  = 0.999 with 0.001 incrementsšSecondly, we calculated the first and second derivatives of the quantile function. The first derivative is also called the sparsity function and corresponds to the reciprocal of the density function. Therefore the second derivative of the quantile function, which measures its curvature, provides an indication of the rate at which data become more sparse (less dense) as the extreme end of the distribution is approached; note that this second derivative takes only non-negative values and increases very quickly for sufficiently high probabilities from models in the exponential family. Thirdly, we identified the take-off point of the curvature, which signals the beginning of the extreme tail of the distribution where the density and sparsity of the data starts decreasing and increasing respectively rapidly. Finally, we defined the EHCV threshold as the upper limit of the one-sided 95% confidence interval for the quantile identified in the previous step. Numerical approximation of the quantile function derivatives was carried out using Hall and Sheather’s [25] bandwidth rule. The large sample size allowed using standard errors obtained with a Powell’s [26] sandwich estimator. The analysis was performed with the R package quantreg [27]. We converted our threshold from 15- to 60-second epochs for ease of comparison with previous studies (all other descriptive statistics are from the 15 second epochs).

Accelerometer data were screened for EHCV and possible error count values including: files containing all five digit values; files containing all one value; count values equal to 2^15^ – 1 = 32,767 which is the maximum value that the ActiGraph 7164 can record regardless of the specified epoch [9], [10]; files containing counts that do not return to baseline [9], [10]; and, negative values [9], [10]. The accelerometer data were also screened to determine whether the metadata corresponded to the pre-defined accelerometer initialization parameters (including date range, date format, activity mode and epoch). Full details on data screening and processing are given in Geraci *et al* [28].

We undertook a sensitivity analysis to compare three different methods of handling EHCV on derived measures of VPA [defined as counts ≥ 3,841 counts per minute (cpm)] using the MCS sample. This cut-off for VPA was taken from a calibration study in seven year olds, carried out specifically for use in the MCS [29]. VPA estimates were derived using the R package pawacc [30]. On all three occasions, children were only included if they had at least two days of data lasting at least 600 minutes per day; periods of greater than 20 minutes of consecutive zero counts were treated as periods of non-wear and excluded [9]. We summarised physical activity, first, including all observations. We then excluded EHCV based on our proposed threshold. Finally, for each child, we excluded EHCV using a threshold proposed by Owen *et al* [15], calculated as the median plus 3.5 times the standard deviation (SD) of all cpm. The estimated SD is considered robust against large deviations as it was obtained by regressing cpm between the 10^th^ and the 90^th^ percentile on their corresponding *z*-scores. Differences between the median daily minutes spent in VPA according to these different methods of excluding EHCV were assessed using the Jonckheere-Terpstra (JT) nonparametric test for homogeneity of medians in a repeated measures setting [31]. We used a Bonferroni-type adjustment for pairwise comparisons, thus the *p*-values should be less than 0.0166 to be considered significant at 5% [32].

## Results

### Sample and accelerometer characteristics

A total of 14,043 children were interviewed at age seven years in the MCS: 13,219 (94.1%) parents/guardians gave consent for their child to wear an accelerometer. A total of 9,005 accelerometer files (70.4% of children consented) were available for analysis following return of accelerometers (excluding those reported as not worn).

### Error count values

A total of 11 (0.12%) MCS children returned accelerometers that contained 237 negative counts comprising 211 counts equal to −32,768 in one child. Overall, negative counts were equal to −32,768 (234 counts), −32,161 (one count), −21,582 (one count), and −9,049 (one count). There were an additional 31 children with files that contained all zero counts and who had presumably not worn the accelerometer. No files were identified that contained all five digit values, all one value (except those with zero counts) or count values that did not return to baseline. An incorrect date format of mm/dd/yyyy was identified in files relating to 22 children, and a further two dates were outside the anticipated range.

### Extreme high count threshold

A total of 860,932,791 count values were measured for the MCS children, of which 777,929,003 (90.4%) were zero (this included night time periods during which the monitors were not worn).The minimum, maximum and quantile distribution of the remaining 83,003,788 non-zero count values are summarised in Table 1.

**Table 1:** Minimum, maximum and quantile distribution of non-zero count values.

Counts per minute									
Minimum	Q1	Q2	Q3	Q90	Q95	Q99	Q995	Q999	Maximum
1	112	452	1378	2753	3765	6530	9036	22271	31695

**Figure 1:**
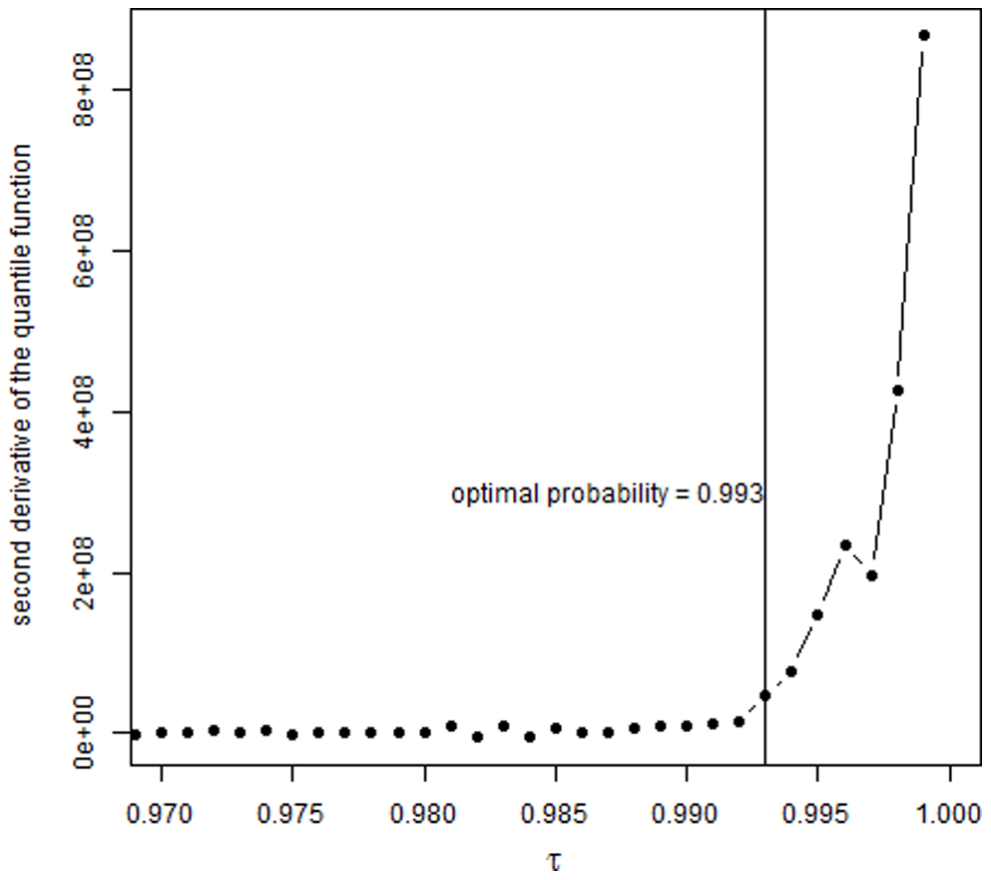
Plot showing the second derivative of the quantile function of non-zero count values.

Changes in curvature of the quantile function defined the optimal probability threshold as 0.993 (Figure 1); this corresponded to a quantile estimate of 1891 counts per 15 second epoch (cpfs) with a standard error of 631 cpfs. To account for estimation uncertainty, we defined the EHCV threshold as the upper bound of the one-sided 95% confidence interval for the quantile 0.993. This was converted to cpm using the formula 4 * (1891+*z_0.95_*×631), where *z_0.95_* is the 95% critical value of a standard normal distribution. Based on this calculation, we propose a count threshold of ≥ 11,715 cpm to define EHCV in large-scale studies in children.

### Extreme high count values

Only 0.7% (581,402 out of 83,003,788) of all non-zero accelerometer counts obtained in MCS children were considered to be EHCV (≥ 11,715 cpm). At least one EHCV was observed in 6459 (71.7%) MCS children: in 8978 (99.7%) and in 2550 (28.3%) <1% and 0% total non-zero counts respectively were classified as EHCV.

### Sensitivity analysis

The EHCV threshold obtained following Owen *et al*’s [15] method was ≥ 19,442 cpm (4,122+3.5×4,377 ). The three estimates of median daily minutes spent in VPA differed significantly according to the method used to exclude EHCV (Table 2; JT *p*<0.0001). Pairwise, median daily minutes spent in VPA were not significantly higher when including all count values compared to using Owen *et al*’s [15] threshold to exclude EHCV (*p* = 0.08). In contrast, median daily minutes spent in VPA obtained using our proposed threshold were significantly lower than those estimated without excluding EHCV (*p* = 0.0001; 6.9 vs. 6.5 minutes per day) and lower than those estimated when excluding EHCV using Owen *et al*’s [15] threshold ( *p* = 0.0104; 6.8 and 6.5 minutes per day). After a Bonferroni-type adjustment for multiple comparisons, conclusions on significance at the 5% level remained unchanged for all pairwise tests.

**Table 2:** Summary statistics and Jonckheere-Terpstra test *p*-values for daily minutes spent in vigorous physical activity using different methods of excluding extreme high count values.

Definition of extreme high count value threshold	Median daily vigorous physical activity (minutes)	Interquartile range daily vigorous physical activity (minutes)
(1) None	6.9	2.1, 12.0
(2) ≥ 19,442 counts/ minute [15]	6.8	2.0, 12.0
(3) ≥ 11,715 counts/ minute	6.5	2.0, 11.0
		
Jonckheere-Terpstra test	*p*-value†	
Global test: (1) > (2) > (3)	<0.0001	
(1) > (2)	0.0814	
(1) > (3)	0.0001	
(2) > (3)	0.0104	

A Bonferroni-type adjustment for multiple comparisons meant that pair-wise *p*-values needed.

to be < 0.0166 to be considered significant at 5%.

## Discussion

### Summary and recommendations for study practice

Based on population-based data, we have developed methodological principles for establishing an EHCV threshold and propose an accelerometer count threshold of ≥ 11,715 cpm be used to define EHCV in accelerometer studies using the ActiGraph GT1M. We have investigated EHCV in order to determine the influence and occurrences of these values in a large scale study of accelerometer-determined PA in children. We found that although EHCV ≥ 11,715 cpm were observed in a high proportion of children (72%) they represented a very small proportion of the total non-zero count values (0.7%). Despite the small proportion of EHCV observed, setting a threshold to distinguish between genuine VPA and those caused by device malfunctioning should be part of any study collected high frequency accelerometer data. Using the proposed EHCV threshold will enhance quality control checks by removing implausible count data and improving the validity of VPA estimates.

We have shown that the method used to define and exclude EHCV can significantly affect estimates of the daily duration of VPA: the median daily minutes spent in VPA was on average 6.2% higher when EHCV were not excluded from analysis compared to estimates obtained using our proposed threshold (≥ 11,715cpm). Similarly, significantly higher (4.6%) estimates were obtained by using a higher threshold (≥ 19,442 cpm) as proposed by Owen *et al* [15]. If researchers do not remove EHCV this may lead to an overestimate of the amount of time children spend in VPA with implications for evidence used to set and assess adherence to guidelines [33]. Although the absolute differences in VPA were small, the significant differences observed may alter associations in multivariable regression models. In addition, these differences may be even more marked if bouts of activity are accumulated to meet such guidelines.

Although unusual, malfunction of the ActiGraph GT1M can result in both negative and positive error count values that were not the same as those observed in the 7164 model. We found error counts were more likely to be recorded as negative values, with −32,768 being the most common. We therefore recommend that data should be screened during fieldwork for the error count values identified so that failing monitors are not re-issued and subsequent data lost.

This study also raises awareness that some manual errors during initialization can occur in studies distributing large numbers of accelerometers. Accelerometer data processing software enables data produced from large-scale studies to be quickly and reliably processed and summarised as, for example, minutes spent each in moderate to VPA. Accelerometer processing software relies on a set of predefined processing criteria that are applied to all data files. Increased efforts are required during initialization so that these predefined parameters are standardized for all study participants so that software can quickly and reliably process the data. It is also advisable for researchers to check these parameters before processing so that incorrectly programmed data are not processed which may result in unreliable summary data. We agree with Matthews *et al* [13] that there is a need to develop standardised quality control procedures prior to data processing in large-scale studies: we suggest that these should include the identification and removal of EHCV, error count values, and incorrectly initialized accelerometers.

### Comparison with existing research

There have been no published reports proposing a predefined count threshold for EHCV in the ActiGraph GT1M based on population-based accelerometer data. Furthermore, few population-based studies have reported the use of a threshold to remove EHCV, and in those that have, the study sample, accelerometer protocols, and thresholds used have varied, with EHCV most commonly removed using a threshold based on basic analytical methods, such as the SD and the median cpm.

Masse *et al* [19] reviewed the methodology of accelerometer-based studies in children published in 2003 and 2004, and reported only one large-scale study that used a threshold to remove EHCV [18]. We have subsequently reviewed the methodology of all (*n*  =  23) large-scale (≥ 250 participants) accelerometer-based studies of children’s (2−18 years) PA published by June 2012 and identified four (13%) that have reported removing EHCV [15], [16], [17], [18], and only one of these used the ActiGraph GT1M [15]. In the European Youth Heart Study, count values greater than 9 SDs away from the median were removed from analyses in data obtained using the ActiGraph 7164 from 2,185 ten and fifteen year olds as these were regarded as representing a ‘distinct unphysiological pattern’ (threshold count value not specified) [18]. The Avon Longitudinal Study for Parents and Children also used the ActiGraph 7164 to collect data from 5,595 twelve year olds, and they removed whole days if the average daily count values were > 3 SDs (1,665 cpm) [16]. In the Child Heart Health Study in England, count values 3.5 SD greater than the median were identified as ‘outliers’ and removed from ActiGraph GT1M data obtained from 2,144 nine to ten year olds [15]. Neither of these studies provided justification for the approach taken to identify EHCV. Using the ActiGraph 7164, Tremblay *et al* used a similar method to remove extremely high counts by removing values that were greater than 3 SD from the mean in their study of 399 eight and 13 year old Canadian children [17]. By using SD, these studies implicitly assume a normal distribution for accelerometer counts; this may not be true, and non-parametric quantile-based methods such as those used in the current study provide a more robust approach.

Within smaller studies, the threshold defined in our study is lower than that proposed by Esliger *et al* (15,000 cpm) using the ActiGraph 7164 [9]. This threshold was obtained by calculating the mean of the maxima count values (11,555 cpm) in 94 eight to 13 year old children. Using this threshold they concluded that values equal to or greater than 15,000 cpm would be very rare in children.

We found one study that used observational data to propose an EHCV threshold, but this used the Actical accelerometer and encompassed a very wide age range. Colley *et al* [34] conducted an extrapolation procedure to propose the threshold, using treadmill speed and corresponding Actical accelerometer counts in 38 Canadians (nine to 59 years) [34]. The study proposed a threshold of ≥ 20,000 cpm for the Actical, which is higher than that proposed by our study using the ActiGraph GT1M. This difference may be due to differences in sample age and size, the different accelerometer model used, and/or the alternative analytical methods used in the current study to determine the threshold.

Few studies measuring PA in children report EHCV recorded by accelerometers. We found fewer records of error count values in the MCS children (0.12%) than reported by the International Children’s Accelerometry Database (ICAD). In the ICAD project, a total of 44,454 ‘viable’ accelerometer files from 20 studies worldwide using either the ActiGraph GT1M or 7164 were processed, with 556 out of 45,190 (1.2%) possible files considered to be ‘spurious’ as they contained at least three consecutive counts at the same value at a count ≥ 10 (with plateaus occurring most often at 32,767); negative values were not reported [10].

Colley *et al* inspected seven days of ActiGraph 7164 data obtained from 987 six to 79 year old Canadians [34]: EHCV were very unusual with almost none exceeding 20,000 cpm. On the rare occasions when excessively high count data (≥28,404 cpm) were identified, the entire recording was found to be completely unusable because of accelerometer malfunction.

The maximum count value recorded in a study using the ActiGraph 7164 was similar (31,346 cpm) to that recorded in this study (31,695 cpm) [9]. We did not find that count values plateaued at 32,767 [9], [10], or at any other high count value in the ActiGraph GT1M, in contrast to reports based on the ActiGraph 7164. However, we did find that the ActiGraph GT1M occasionally malfunctioned by recording negative values, in particular a count value of −32,768. The ActiGraph GT1M is a 16-bit machine with the potential to store and record signed values from −32,768 to 32,767 (±2^15^−1): count values as high 32,767 may not be recorded because of an enhanced digital filter that was not incorporated in the 7164 model. The GT1M also has an improved capacity to measure at higher frequencies than the 7164 because it filters acquired data within a frequency range of 0.25 to 2.5 Hz, compared to 0.21 to 2.28 Hz in the ActiGraph 7164 [35].

No other published study has compared different approaches to excluding EHCV on estimates of VPA. Research has shown that VPA is associated with positive health benefits independently of overall PA [37], with one study reporting that more than 7 minutes of VPA is associated with a reduced risk of overweight and elevated blood pressure [36]. Our study found small absolute differences in the time spent in VPA when different methods to exclude EHCV were used, but the proportional differences were much larger (6.2%). An evidence-based recommendation for the duration of time children should spend in VPA has not yet been made but is likely to rely on evidence from accelerometer-based studies [33]. It is therefore important that such studies adopt a consistent approach to identifying and excluding EHCV as we have shown that this may introduce significant and systematic biases in estimates of VPA.

### Strengths and limitations

This is the first study to propose a predefined EHCV threshold in the ActiGraph GT1M using population-based accelerometer data. The novel analytical methods employed here enable a robust threshold to identify EHCV to be defined. Approaches to measuring the distance of the observations from the centre (e.g. the mean or the median) of the distribution are typically based on the notion of some underlying probability model (e.g., the ‘three-sigma’ rule using the normal distribution) which might mislead the analysis. In contrast, non-parametric techniques that focus on the tails of the distribution provide a means to assess the local behaviour of the data in the region of interest, and to quantify the amount of information that can be reasonably excluded from the analysis. We used distribution-free quantile-based methods as opposed to parametric methods as they allow for characterisation of the distribution of the counts without making untenable assumptions.

This research is of interest because it analyses an aspect of accelerometry that is rarely discussed; EHCV are uncommon and analysing them empirically with confidence requires large datasets. Uniquely, we have applied this threshold in a large population-based accelerometer study to determine frequencies of EHCV, and the influence of varying this threshold in order to exclude implausible count values on estimates of VPA. We have also identified typical error count values recorded by the ActiGraph GT1M. In doing so, we used data from a large, contemporary, socially and ethnically diverse cohort of seven year old children, from all four UK countries.

As this study concerns EHCV which result from accelerometer malfunction or misuse rather than plausible human activity, our methodology should be applicable for use in all ages. Even during very extreme running speeds (up to 20 kilometres/hour^1^) the Actigraph GT1M has only been shown to record a maximum of 9170 (±971) cpm in adult males [37]. However, our proposed threshold value is specific to the ActiGraph GT1M and may not be applicable in different accelerometer models. Accelerometers vary in cost, feasibility, validity and reliability; thus each model may require a different threshold value to define EHCV. Future research should be aimed at determining whether the EHCV threshold proposed by the current study is applicable in different accelerometer models. The statistical methods can be employed in other studies using different accelerometer models, and can also be employed in other medical devices that yield high frequency values.

## Conclusion

Accelerometer data collection is normally completed before any data quality control checks can be undertaken. Although uncommon, accelerometer malfunction can occur; studies therefore need to check for error count values throughout fieldwork so that faulty monitors are not re-issued. It is also crucial for population-based studies to integrate a core set of quality control procedures prior to data processing, including the identification of EHCV, typical error count values, and incorrectly initialised accelerometers. Using a count threshold of ≥ 11,715 cpm will enhance data cleaning in studies collecting ActiGraph GT1M -determined measurements of PA in children by ensuring only plausible data are analysed, thus improving the validity of VPA estimates. Studies using different accelerometer models can use our robust methodological protocol for establishing their own EHCV threshold.
